# Multiple Intraventricular Neurocysticercal Cysts Treated with Endoscopy without Antiparasitic Therapy

**DOI:** 10.4269/ajtmh.2011.10-0731

**Published:** 2011-07-01

**Authors:** Theodoros Kelesidis, Nguyen Thian

**Affiliations:** Department of Medicine, Division of Infectious Diseases, David Geffen School of Medicine at University of California Los Angeles, Los Angeles, California; Department of Surgery, University of California School of Medicine, Los Angeles, California

Neurocysticercosis is of emerging importance in the United States because of immigration from disease-endemic regions of Latin America.^1^ Intraventricular neurocysticercal cysts are probably more frequent than previously thought and usually occur as single cysts in the fourth ventricle or as multiple cysts that frequently coexist with parenchymal and sub-arachnoid cysts.^2^ However, multiple intraventricular cysts without accompanying visible parenchymal cysts are a relatively uncommon manifestation of neurocysticercosis, and it remains unclear if endoscopic removal of these cysts is adequate without antiparasitic therapy.

**Figure 1.**
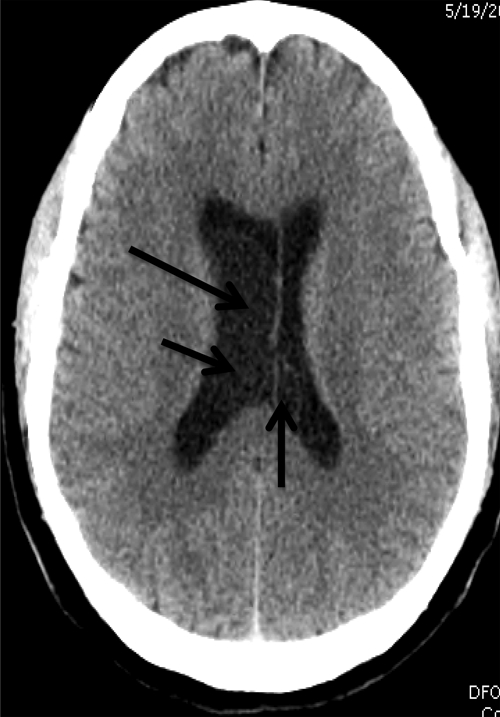
Computed tomography of the head of the patient without contrast showing two right intraventricular and one left intraventricular cystic lesions.

**Figure 2.**
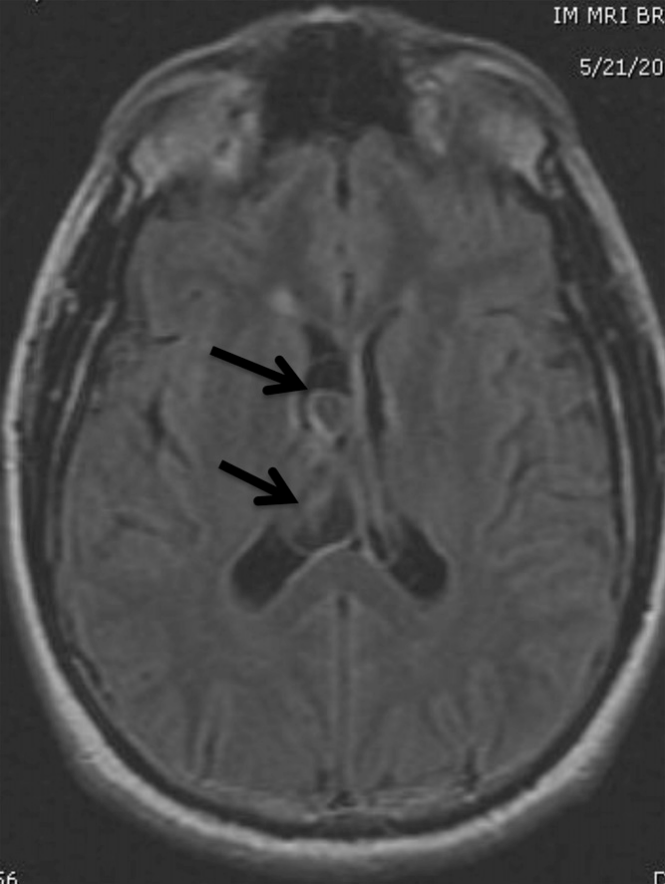
Magnetic resonance imaging of the brain (transverse view) of the patient showing two right intraventricular and one left intraventricular cystic lesions at varying stages. There is a larger 2.3-cm lesion in the posterior aspect of the right ventricle, which follows fluid signal on all sequences. There is subtle mass effect of the right ventricle, which appears asymmetrically enlarged compared with the left ventricle. There is no obstructing lesion in the region of the foramen of Monro or third ventricle. No parenchymal lesions were identified.

**Figure 3.**
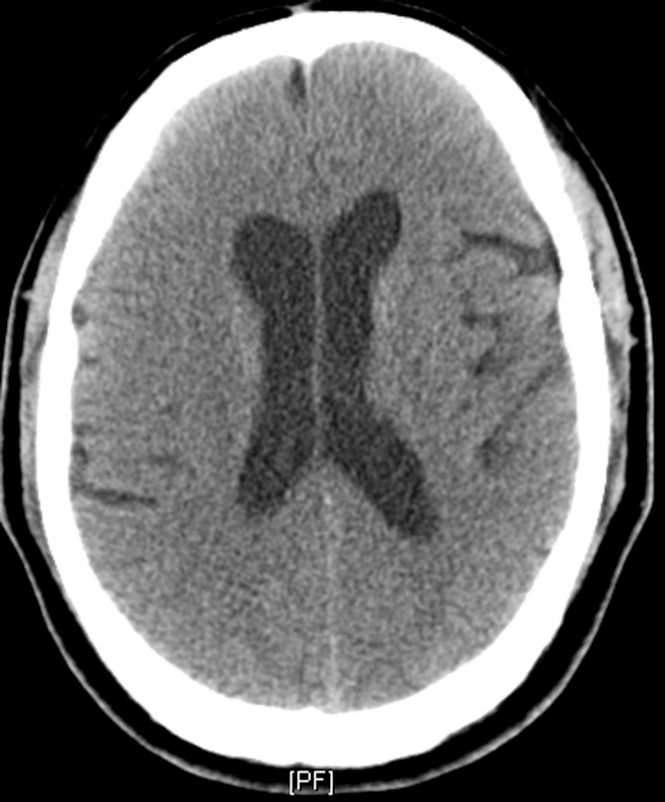
Computed tomography of the head of the patient without contrast status post removal of intraventricular cysticercal cysts.

A 41-year-old man (Mexican immigrant) had headache of two-months duration. Results of brain imaging and immunologic studies were suggestive of intraventricular neurocysticercosis and three cysts were identified (Figures 1 and 2). There were no parenchymal cysts. Surgical removal of the cysts with flexible endoscopy resulted in resolution of symptoms. The patient received corticosteroids for three weeks but no antiparasitic treatment. After one-year follow-up, he remained asymptomatic without any cysts when examined by repeat neuroimaging (Figure 3).

Intraventricular neurocysticercosis may have a risk of ependymitis in those treated with antihelminthic drugs.^2^This case illustrates that multiple intraventricular cysts in the absence of parenchymal cysts can be treated with endoscopic removal without concurrent use of antiparasitic agents at centers having the required expertise, particularly if cysts are large. Endoscopical removal of these cysts in the absence of visible cysts on repeat imaging after one year may indicate presumptive cure. However, we cannot conclude that the patient had definite long-term cure because small cysts may show up years later and one-year follow-up does not rule out this possibility. Thus, long-term follow-up imaging with monitoring of serum serologic results for neurocysticercosis is recommended.
